# Cranial arterial pattern of the Sri Lankan spotted chevrotain, *Moschiola memmina*, and comparative basicranial osteology of the Tragulidae

**DOI:** 10.7717/peerj.1451

**Published:** 2015-12-01

**Authors:** Haley D. O’Brien

**Affiliations:** Departement of Biological Sciences, Ohio University, Athens, OH, United States of America; Department of Anatomy and Cell Biology, Oklahoma State University College of Osteopathic Medicine, Tulsa, OK, United States of America

**Keywords:** Artiodactyla, Tragulidae, Carotid rete, Cranial vasculature, Ruminant, Evolution, Anatomical imaging

## Abstract

The cranial arterial pattern of artiodactyls deviates significantly from the typical mammalian pattern. One of the most striking atypical features is the rete mirabile epidurale: a subdural arterial meshwork that functionally and anatomically replaces the arteria carotis interna. This meshwork facilitates an exceptional ability to cool the brain, and was thought to be present in all artiodactyls. Recent research, however, has found that species of mouse deer (Artiodactyla: Tragulidae) endemic to the Malay Archipelago possess a complete a. carotis interna instead of a rete mirabile epidurale. As tragulids are the sister group to pecoran ruminants, the lack of a rete mirabile epidurale in these species raises intriguing evolutionary questions about the origin and nature of artiodactyl thermoregulatory cranial vasculature. In this study, cranial arterial patterns are documented for the remaining species within the Tragulidae. Radiopaque latex vascular injection, computed tomography (CT-scanning), and digital 3-dimensional anatomical reconstruction are used to image the cranial arteries of a Sri Lankan spotted chevrotain, *Moschiola meminna*. Sites of hard and soft tissue interaction were identified, and these osteological correlates were then sought in nine skulls representative of the remaining tragulid species. Both hard and soft tissue surveys confirm that the presence of an a. carotis interna is the common condition for tragulids. Moreover, the use of a 3-D, radiographic anatomical imaging technique enabled identification of a carotico-maxillary anastomosis that may have implications for the evolution of the artiodactyl rete mirabile epidurale.

## Introduction

Among living ruminant artiodactyls, perhaps the most enigmatic clade is the family Tragulidae. Commonly known as mouse deer or chevrotains for their exceptionally small size, Tragulidae is one of the least diverse groups within the artiodactyl suborder Ruminantia (*sensu* Spaulding, O’Leary & Gatesy, 2009), containing only 3 genera: *Tragulus* and *Moschiola* native to South East Asia, and the single African genus *Hyemoschus* (*sensu* Rössner, 2007). Ranging in length from 45 to 80 cm and 1.5 to 15 kgs in mass, tragulids are the smallest hoofed mammals alive today. When compared to the majority of ruminants, tragulids can be characterized by a number of primitive behaviors and traits. They are, for example, largely nocturnal, lack cranial appendages, and possess a 4-chambered stomach with rudimentary subdivisions and a greatly reduced omassum (Milne-Edwards, 1864; Carlsson, 1926; Langer, 1974; Dubost, 1975). Biogeographically, they can be found in relict, disjunct populations in the tropical forests of Southeast Asia and West Africa (Grubb, 1993). This suite of primitive traits (detailed in Rössner, 2007) and relict distribution have led to the designation of the group as “living fossils” (Janis, 1984). Because of this, tragulids are commonly used as a model for early ruminant evolution (Métais & Vislobokova, 2007).

**Figure 1:**
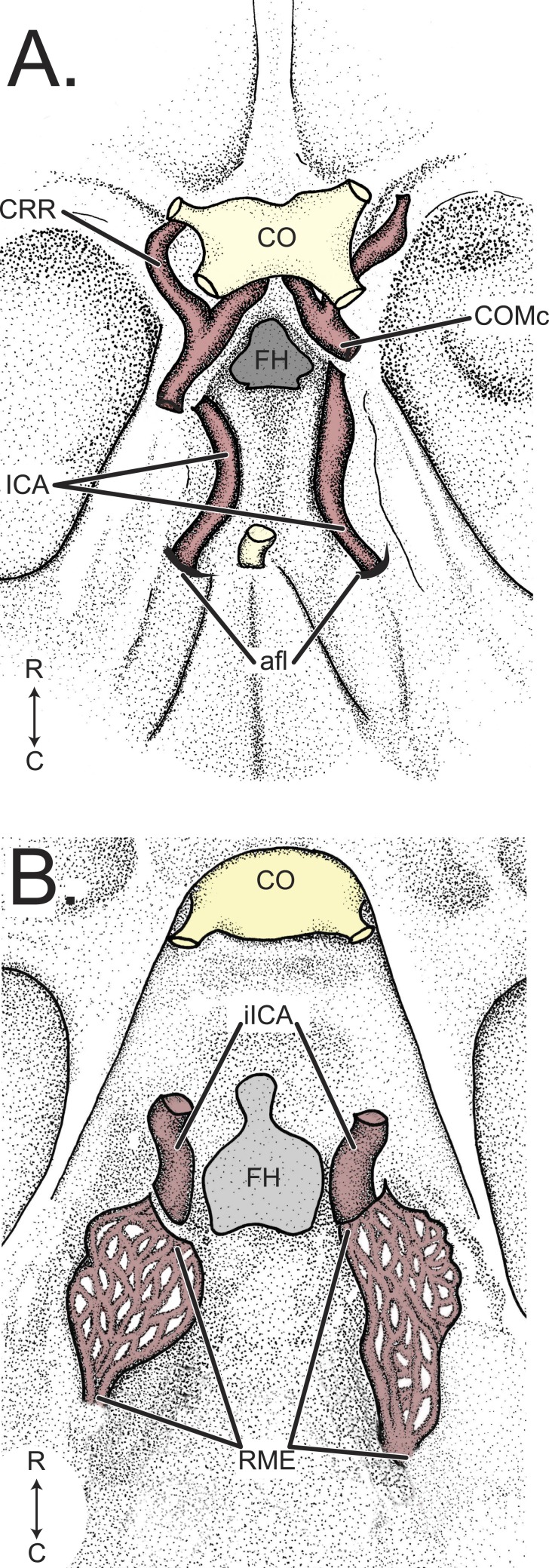
Comparison of *Tragulus* and *Capra* arteries of the cranial base, internal surface. (A) The cranial arterial supply of the lesser mouse deer, *Tragulus javanicus*, derives from a large, tubular a. carotis interna which enters the braincase through the anterior foramen lacerum and courses rostrally along the internal surface of the os basisphenoidale. (B) The cranial arterial supply of the domestic goat, *Capra hircus*, derives from a rete mirabile epidurale. The rete is supplied by the a. maxillaris rostrally and the a. pharyngea ascendens caudally. The rete fills the floor of the basicranium and contributes the pars intracranialis a. carotis interna to the circulus arteriosus cerebri. *Abbreviations*—afl, anterior foramen lacerum; CO, chiasma opticum; COMc, arteria communicans caudalis (caudal loop of the circulus arteriosus cerebri); CRR, arteria cerebri rostralis (rostral loop of the circulus arteriosus cerebri); FH, fossa hypophysialis; ICA, arteria carotis interna; iICA, pars intracranialis arteria carotis interna; RME, rete mirabile epidurale.

Corroborating the notion that tragulids are living fossils is the apparent lack of a rete mirabile epidurale in the lesser and greater mouse deer, *Tragulus javanicus* and *T. napu*, respectively (Fig. 1A; Fukuta et al., 2007). The rete mirabile epidurale is a thermoregulatory cranial arterial meshwork that enables artiodactyls to have one of the most advanced capacities for selective brain cooling (reviewed by Caputa, 2004). This structure provides the predominant supply of oxygenated blood to the brain by anatomically and functionally replacing the a. carotis interna, such that in mature individuals, only the pars intracranialis of the a. carotis interna remains (Fig. 1B; Daniel, Dawes & Prichard, 1953). Due to the combined effects of the rete’s high surface area and the reservoir of maxilloturbinate-cooled blood in the rete’s cavernous venous sinus housing, heat is rapidly transferred from arterial blood destined for the brain into to the venous blood that returns to the trunk (Jessen, 1998). As cooled blood flows directly to the hypothalamus, this dissipation of heat is an effective mechanism for delaying heat stress and evaporative water loss, thereby conserving water (Jessen & Pongratz, 1979; Kuhnen, 1997; Jessen, 1998; Mitchell et al., 2002; Ostrowski, Williams & Ismael, 2003). Prior to the study conducted by Fukuta and colleagues (2007), all artiodactyls were thought to possess this advantageous structure. The absence of a rete in the genus *Tragulus* may be the result of (1) their exceptionally small body size, (2) the basal status of the Tragulidae among ruminants, or (3) an adaptation to their unique ecology. Small body size is an unlikely explanation for the tragulid cranial arterial pattern, as other small-bodied ruminants possess a rete (Fukuta et al., 2007). Moreover, scaling functions for continuous endothermy (McNab, 1983) and evaporative water loss (Altman & Dittmer, 1968) indicate that smaller-bodied mammals have a relatively *higher* thermoregulation-specific metabolic rate and require more water on a mass-specific basis (McNab, 1990). With body size an unlikely explanation, investigations into the evolutionary hypotheses of plesiomorphy or apomorphy may help shed light on why tragulids have an a. carotis interna in lieu of a rete mirabile epidurale. These studies are, however, hampered by a lack of comparative anatomical data.

In this study, the cranial arterial pattern of a second genus of mouse deer, *Moschiola*, is described in order to confirm the aberrant tragulid cranial arterial pattern outside of *Tragulus*. Next, this data was used to identify osteological correlates for tragulid cranial arteries that could be sought in a phylogenetically comprehensive sample of much more readily available skulls. Through this survey of hard and soft tissue cranial morphology, it can be concluded that the absence of a rete mirabile epidurale is common to extant tragulid species. Furthermore, the use of modern anatomical data collection methods enabled the identification of a possible evolutionary mechanism underlying the presence of the rete mirabile epidurale in the derived ruminant condition.

## Materials and Methods

### Soft tissue data collection

A single individual of *Moschiola meminna* was obtained on loan from the American Museum of Natural History Department of Mammalogy (AM-201747). Tragulids are rare in the wild (Rössner, 2007), and utilization of an alcohol-preserved specimen was the most feasible option for obtaining a specimen. Due to the terms of the specimen loan, physical dissection was not performed in order to maintain the integrity of the specimen for future study. Soft tissue data collection therefore followed non-destructive digital dissection methods.

The left and right arteria carotis communis were dissected, low in the neck, from the whole-body specimen of *Moschiola*, and the left arteria carotis communis was cannulated with an 18-gauge angiographic cannula (Beckton-Dickinson, Worldwide). The cannula was fixed in place with surgical ligature and adhesive. The right a. carotis communis was ligated as a pressure-outlet and means of monitoring injection progress (O’Brien & Williams, 2014). To clear coagulated blood from the vascular tree, the arterial system was manually flushed with warm water for 10 min, followed by perfusion with 90 mL of 40% One-Point anticoagulant solution. A 10% concentration of anticoagulant solution is recommended for most injections, however, due to the long-term storage of the specimen (over a century), a higher concentration of 40% was used in effort to break up large blood clots. Following initial specimen preparation, radiopaque latex vascular injection was conducted, following the protocol of Holliday et al. (2006) and the perfusion criterion of O’Brien & Williams (2014). The specimen was injected with 5 mL of 40% Liquid Polibar Plus barium sulfate suspension (BaSO_4_, E-Z-Em, Westbury, NY, USA) in red liquid latex injection medium (Ward’s, Rochester, NY, USA). Perfusion continued until latex emerged from the contralateral a. carotis communis. Acetic acid (10% glacial acetic acid solution) was used to set any extravasated latex.

**Figure 2:**
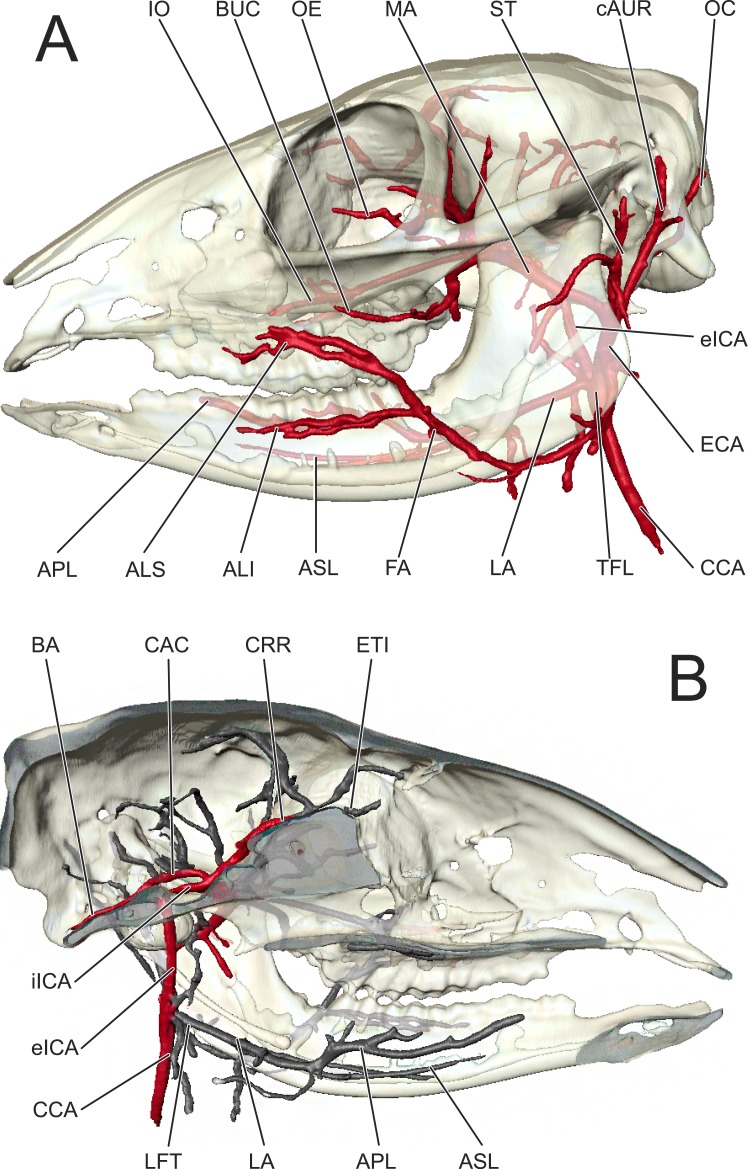
Cranial arteries of *Moschiola memmina*. (A) Left lateral view; (B) ‘Sagittal section, left medial view. *Abbreviations*—ALI, arteria labialis inferior; ALS, arteria labialis superior; APL, arteria profunda linguae; ASL, arteria sublingualis; BA, arteria basilaris; BUC, arteria buccalis; CAC: circulus arteriosus cerebri; cAUR, arteria auricularis caudalis; CCA, arteria carotis communis; CRR, arteria cerebri rostralis; ECA, arteria carotis externa; eICA, arteria carotis interna pars extracranialis; ETI, arteria ethmoidalis interna; IO, arteria infraorbitalis; FA, arteria facialis; iICA, arteria carotis interna pars intracranialis; LA, arteria lingualis; MA, arteria maxillaris; OC, arteria occipitalis; OE, arteria ophthalmica externa; ST, arteria temporalis superficialis; TFL, truncus linguofacialis.

**Figure 3:**
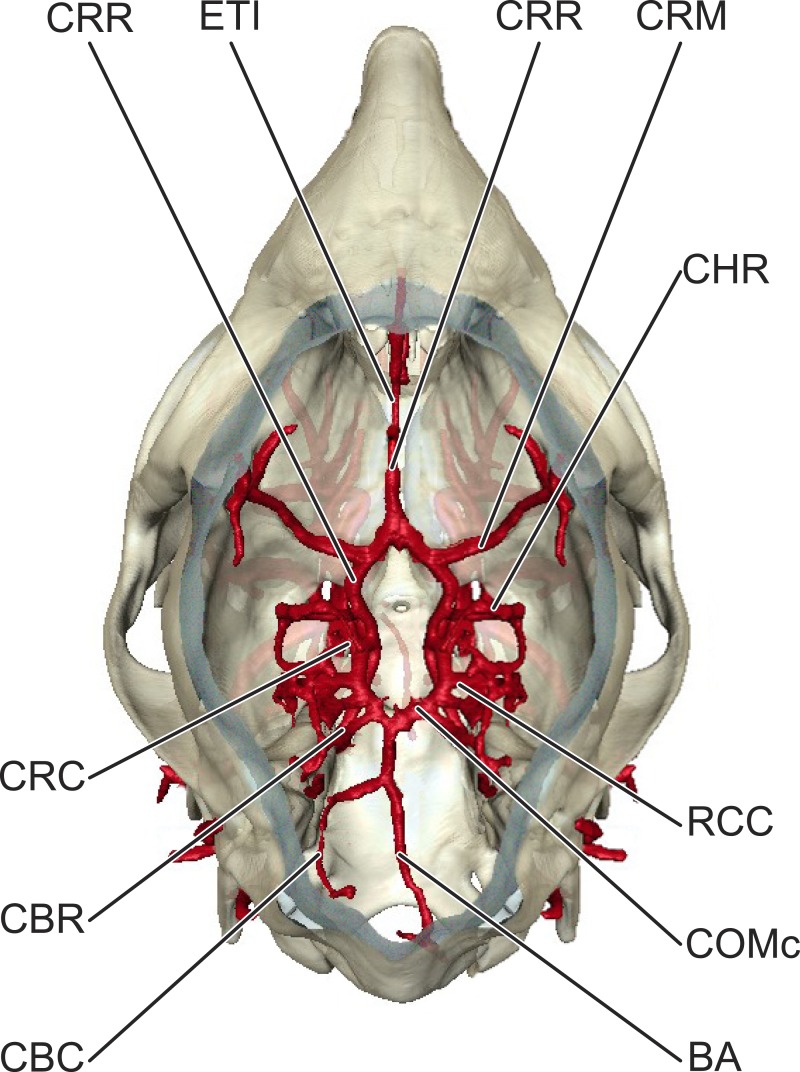
Cerebral arterial circle of *Moschiola memmina*. Dorsal view of the circulus arteriosus cerebri, *in situ*. *Abbreviations*—BA, arteria basilaris; CBC, arteria cerebelli caudalis; CBR, arteria cerebelli rostralis; CHR, arteria choroidea rostralis; COMc, arteria communicans caudalis; CRC, arteria cerebri caudalis; CRM, arteria cerebri media; CRR, arteria cerebri rostralis; ETI, arteria ethmoidalis interna.

Following radiopaque latex injection, the specimen was CT scanned at the Holzer Clinic in Athens, Ohio, on a Philips Brilliance 64-slice CT scanner. By scanning at 0.67 mm slice thickness, 150 kV, and 80 mA, a voxel size of 0.693359 × 0.693359 × 0.5 mm was achieved. The resultant data were up-sampled to a size of 0.1 × 0.1 × 0.1 mm during post-processing in Avizo (version 7.0; VSG). Up-sampling does not affect the inherent quality of the data, and is a reliable technique for generating a visually smoother surface upon reconstruction. Because a 40% barium concentration yields stark contrast between hard tissues, the skull and arteries were segmented based on distinctive gray-scale values. Manual segmentation was then employed to verify and edit the accuracy of the model. Segmented morphology was then rendered in three dimensions (Figs. 2 and 3; http://figshare.com/s/e22fde06688711e5babe06ec4bbcf141; http://figshare.com/s/f1f3cb9a688711e5ab6506ec4bbcf141).

### Hard tissue data collection

Skulls of mature individuals representing the four conventional species of tragulids were examined for osteological correlates (Table 1; for taxonomy see discussion in Rössner, 2007). Presence or absence of each correlate was entered into a character matrix using Mesquite (v. 2.75, Maddison & Maddison, 2011; Table 1). The cranial osteology of tragulids was compared with artiodactyl species known to possess a rete mirabile epidurale, including the domestic goat (*Capra hircus*) and the white-tailed deer (*Odocoileus virginianus*), as well as the domestic horse, *Equus ferus caballus*, a perissodactyl with an a. carotis interna (Table 1).

**Table 1:** Specimens examined and osteological correlates. In addition to mature, adult specimens, specimens of sub-adult, early juvenile, and neonate tragulids were examined to account for ontogenetic changes in osteology. The following character states were used: *Carotid groove, os basisphenoidale*—Character state [1]: sulcus caroticus present on the internal surface of the os basisphenoidale. Correlates to presence of the a. carotis interna. Character state [0]: internal surface of the os basisphenoidale is smooth medial to the lateral sellar compartment, and lacks a sulcus caroticus. Correlates to presence of the rete mirabile epidurale. *Medial bullar groove*—Character state [1] sulcus caroticus present on the medial surface of the bulla tympanica. Correlates with presence of the a. carotis interna. Character state [0]: sulcus caroticus absent on the medial surface of the bulla tympanica. Correlates with presence of the rete mirabile epidurale. *Foramen orbitorotundum*—Character state [1]: ventromedial notch in the wall of the presphenoid/foramen orbitorotundum is present. Correlates with a tubular a. maxillaris and indicates the presence of an a. carotis interna. Character state [0]: foramen orbitorotundum un-notched. Correlates with the diffuse rami that supply the rete mirabile epidurale from the a. maxillaris and indicates the presence of the rete mirabile epidurale. *Foramen lacerum*—Character state [1]: the foramen lacerum is notched due to the presence of an a. carotis interna. Character state [0]: the foramen lacerum is absent or un-notched, indicating the absence of an a. carotis interna. *Foramen ovale*—Character state [1]: foramen ovale is notched and/or trapezoidal in contour. Correlates with a. maxillaris ramus anastomoticus that traverse the foramen ovale to ramify the rete mirabile epidurale. Character state [0]: foramen ovale is un-notched and oval in contour. Correlates with the absence of rami supplying the rete mirabile epidurale.

Specimen information			Osteological correlates					
Taxon	Specimen no.	Approximate age	Carotid groove: BSPH	Medial bullar groove	Foramen orbitorotundum	F. orbitorotundum: ventromedial notch	For. lacerum: notched	For. ovale: notched
*Hyemoschus aquaticus*	AM-89414	Adult	1	1	1	1	1	0
*Hyemoschus aquaticus*	AM-146844	Adult	1	1	1	1	1	0
*Hyemoschus aquaticus*	CM-2692	Adult	1	1	1	1	1	0
*Hyemoschus aquaticus*	AM-53634	Sub-adult	1	1	1	1	1	0
*Tragulus javanicus*	AM-106302	Sub-adult	1	1	1	1	1	0
*Tragulus javanicus*	AM-107113	Neonate	0	0	1	0	0	0
*Tragulus javanicus*	AM-188310	Early Juvenile	1	0	1	0	0	0
*Tragulus napu*	CM-88224	Adult	1	1	1	1	1	0
*Moschiola memmina*	AM-201747	Adult	1	1	1	1	1	0
*Odocoileus virginianus*	OUVC	Adult	0	0	1	0	0	1
*Capra hircus*	OUVC	Adult	0	0	1	0	0	1
*Equus ferus caballus*	OUVC	Adult	1	–	0	–	1	0

Osteological correlates are defined as a “causal relationship” between soft tissues and their surrounding osteology (Witmer, 1995), so the ontogeny and homology of the a. carotis interna were taken into account. The embryonic development of cranial vasculature in *Tragulus javanicus* has been detailed by Wible (1984). Because skull development continues post-partum, skulls of *Tragulus javanicus* (3) and *Hyemoschus aquaticus cottoni* (1) neonates and juveniles were obtained on loan from the American Museum of Natural History Department of Mammalogy. These specimens ranged in relative age from neonate (AM-107113) and early juvenile (AM-188310) to sub-adult (AM-106302 and AM-53634), as approximated by extent of cartilage, degree of suture closure, and skull length (as in Powers, 1962; Morris, 1972; Mitchell, 1973; Meindl & Lovejoy, 1985; Brunner, 1998; Sanchez-Villagra, 2010).

## Results

### Soft tissue results: cranial arterial patterns of *Moschiola meminna*

#### Major distribution of the carotid arteries

In *Moschiola*, the arteria carotis communis (CCA) ascends through the neck and bifurcates into substantial arteria carotis externa and interna (ECA and ICA, respectively) approximately 1 cm caudal to the apparatus hyoideus (Fig. 2). In other ruminants, the a. carotis communis continues as the ECA and the pars extracranialis of the ICA obliterates throughout early ontogeny. As expected for non-artiodactyl mammals, the ICA ascends without branching to the foramen lacerum, through which the vessel enters the braincase (Fig. 2). Immediately after passing through the foramen lacerum, the ICA courses rostrally over the internal surface of the os basisphenoidale (Fig. 3), leaving deep arterial grooves. The pars intracranialis of the ICA then distributes branches to the circulus arteriosus cerebri.

The a. carotis externa gives rise to a number of distributing vessels. At the level of its bifurcation with the ICA, the ECA gives off the rostrally-coursing arteria lingualis, the laterally-coursing arteria facialis (these vessels arise together from a short truncus linguofacialis), and two large rostral and caudal terminations—the arteria maxillaris (MA) and the arteria temporalis superficialis, respectively (Fig. 2). Near its departure from the ECA, the a. temporalis superficialis gives rise to the arteria auricularis caudalis, such that these two vessels share a very short common trunk (Fig. 2). The a. auricularis caudalis then ascends close to the caudal border of the meatus acusticus externus, superficial to the processus jugularis (Fig. 2). Smaller branches arise from the a. auricularis caudalis that distribute to the mastoid and occipital regions until the artery terminates on the dorsal surface of the os temporale pars squamosa. The pinna of the tragulid ear is small in relative and absolute terms, and the latex failed to perfuse the cartilaginous and soft tissue portions of the ear, perhaps due to the reduced caliber of arteries that supply this region. The a. stylomastoideus is a small lateral branch of the a. auricularis caudalis that enters the foramen stylomastoideus, courses through the canalis facialis, and ramifies the middle ear.

The a. temporalis superficialis is the dorsal termination of the ECA. This artery follows the caudal border of the ramus mandibulae toward the processus temporalis of the os zygomaticum (Fig. 2). The largest branch of the a. temporalis superficialis is the arteria transversa faciei, which courses rostrally over the m. masseter (Fig. 2). Smaller muscular branches perforate the caudal portion of the m. masseter and m. temporalis. A minute a. auricularis rostralis splits from the a. temporalis superficialis before the parent artery terminates within the m. temporalis. The a. transversa faciei crosses the caudal border of the ramus mandibulae ventral to the processus condylaris (Fig. 2). The a. transversa faciei does not supply the articulatio temporomandibularis as is common among non-tragulid ruminants.

#### Superficial arteries of the face and scalp

The a. facialis branches from the truncus linguofacialis somewhat higher than the arteria lingualis (Fig. 2). Near its origin, the a. facialis distributes several branches to the glandulae parotis and deeper parenchyma caudal and lateral to the border of the ramus mandibulae. The artery then crosses over the margo ventralis of the corpus mandibulae to course dorsally, superficial to the parotid region. The a. facialis terminates as the a. labialis inferior and the a. labialis superior. The a. labialis inferior, in turn, has multiple branches, both superficial and deep, as it courses superficial to the mandibular dentition to the inferior labium. The a. labialis superior parallels the maxillary dentition, ultimately dispersing within the muscle, skin, and mucosa of the superior labium.

The arteria occipitalis departs directly from the medial surface of the ECA, etching a groove along the posteromedial surface of the bulla tympanica as it ascends. Unlike the a. auricularis caudalis, the a. occipitalis passes medial to the processus jugularis (Fig. 2). The a. occipitalis gives off arteria condylaris and meningea caudalis branches before distributing over the occipital region. The a. meningea caudalis passes through the mastoid foramen to supply oxygenated blood to the caudal meninges.

#### Branches and distribution of the maxillary artery

The a. maxillaris is the largest rostral termination of the ECA, and as is common to other ruminants, supplies the brain, orbit, palate, nasal vestibule, and the superficial and deep structures of the face dorsal to the maxillary dentition and labium. In this specimen of *Moschiola*, which is both over a century old and preserved in alcohol, several branches of the a. maxillaris common to other ruminants did not perfuse with latex. These include the a. auricularis profunda, the a. tympanica rostralis, the a. masseterica, and the a. palatina descendens and a. sphenopalatina. As with the a. auricularis caudalis, the minute pinna of the tragulid auricle renders these arteries less likely to perfuse due to their small internal diameter. The a. tympanica is likewise expected to be small.

The first main branch of the a. maxillaris is the arteria alveolaris inferior. From the ventral surface of the a. maxillaris, the a. alveolaris inferior courses toward and enters the foramen mandibularis. The a. alveolaris inferior did not perfuse past the foramen mandibularis and into the canalis mandibulae.

**Figure 4:**
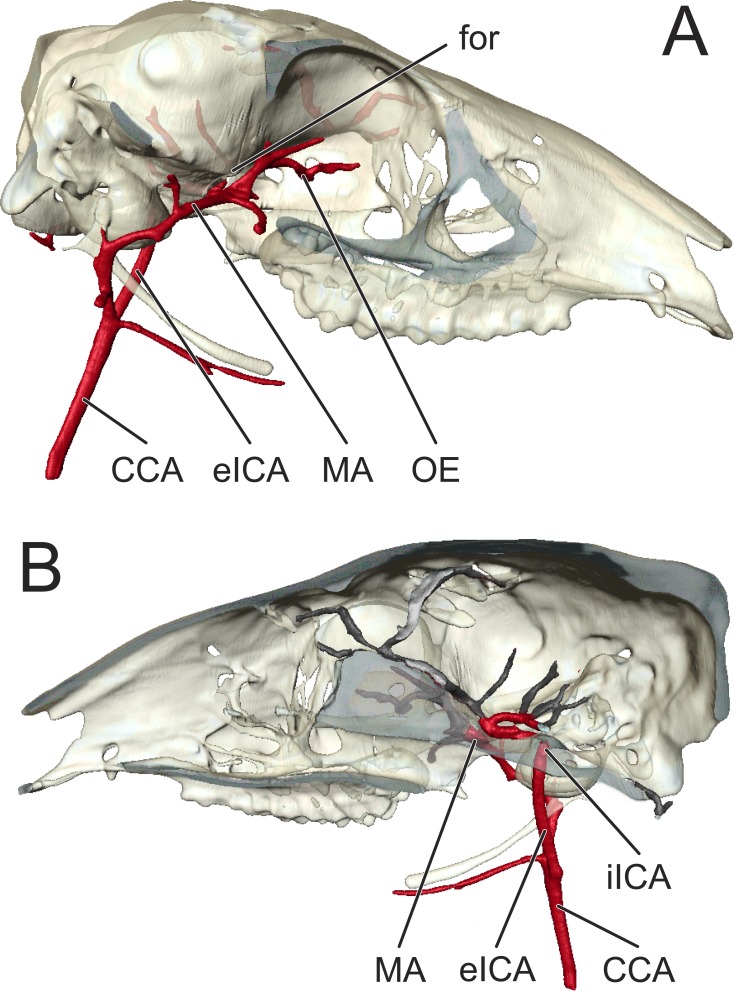
Simplified arterial tree of *Moschiola memmina*, illustrating carotid-maxillary anastomosis. (A) Right lateral view, illustrating the a. carotis interna ascending to the basicranium without branching, and a large ramus from the a. maxillaris entering the foramen orbitorotundum. (B) Right medial sagittal section. The a. carotis interna enters the braincase through the anterior foramen lacerum, and courses rostrally to anastomose with the a. maxillaris. *Abbreviations*—CCA, arteria carotis communis; eICA, arteria carotis interna pars extracranialis; for, foramen orbitorotundum; iICA, arteria carotis interna pars intracranialis; MA, arteria maxillaris; OE, arteria ophthalmica externa.

Following the a. alveolaris inferior and the small arteries that supply the musculi pterygoidei, m. constrictors pharynges rostrales, and mucosae pharyngeus, the a. maxillaris gives off a large branch from its dorsal surface directly into the foramen orbitorotundum. Upon entering the braincase, this artery immediately anastomoses with the a. carotis interna (Figs. 2 and 4). Proximal to the orbital portion of the a. maxillaris arise the arteriae temporalis profunda caudalis and rostralis. The a. temporalis profunda caudalis courses superiorly, deep to the rostral edge of the processus coronoideus. This artery is the primary contributor of blood to the m. temporalis. The a. temporalis profunda rostralis arises shortly after the division of the a. temporalis profunda caudalis, and courses caudal to the processus zygomaticus of the os frontale.

In this specimen, radiopaque latex perfused only three major vessels extending from the rostral extent of the a. maxillaris: the a. ophthalmica externa, the a. infraorbitalis, and the a. buccalis. The a. ophthalmica externa is the dorsal continuation of the a. maxillaris in the orbit (Fig. 2). This artery traverses the periorbita, supplying the m. levator anguli oculi medialis as it travels toward the os ethmoidale. The injection medium did not perfuse the rostral termination of the artery. The a. buccalis is a major lateral branch of the a. maxillaris. The a. buccalis supplies the superficial face by crossing the tuber maxillae. Once it becomes superficial, the a. buccalis courses rostrally, paralleling the crista facialis (Fig. 2). Throughout its course, the a. buccalis supplies the ventral extraorbital region, the musculi pterygoidei, the m. buccinator, and the deep surface of the m. masseter. It does not, however, give rise to the a. temporalis profunda rostralis, as in many ruminants. The a. infraorbitalis is the rostral continuation of the a. maxillaris (Fig. 2). After coursing underneath the eye and periorbita, the a. infraorbitalis enters the canalis infraorbitalis. The maxillary dentition is supplied by the rami dentales before the artery exits the canal through the foramen infraorbitalis.

#### Blood supply to the eye and orbit

The a. ophthalmica interna contributes blood supply to the globe of the eye. Originating on the rostral face of the circulus arteriosus cerebri, the a. ophthalmica interna exits the braincase through the midline canalis opticus and is transmitted to the eye via the nervus opticus. The a. ophthalmica externa, as described above, perfuses the medial and inferior periorbita, inclusive of the extraocular muscles (Fig. 2). Smaller branches supplying the eye and orbit, such as the a. centralis retinae, the a. lacrimalis, and the ae. ciliares, were not perfused.

#### Arterial blood supply to the brain

The a. carotis communis bifurcates into the ECA and ICA ∼1 cm ventral to the hyoid. The ICA ascends toward the basicranium without branching (Figs. 2 and 4). Before entering the braincase through the foramen lacerum, this vessel scours the medial surface of the bulla tympanica, leaving a noticeable groove. This groove is not present in extant artiodactyls with a rete mirabile epidurale. Once inside the braincase, the ICA courses rostrally along the internal surface of the corpus of the os basisphenoidale, again leaving deep grooves (Fig. 3). The circulus arteriosus cerebri (CAC) forms from the anastomosis of the ICA and the a. maxillaris (Figs. 3 and 4). The arteria cerebri rostralis forms the rostrolateral quadrant of the CAC, and continues rostrally in the median plane. Coursing along the nervus olfactorius, the a. cerebri rostralis terminates as rami corticales to the fissure longitudinalis of the cortex cerebri and bulbus olfactorius. The pars rostralis of the cortex cerebri, the nervus olfactorius, and the bulbus olfactorius are supplied by the a. cerebri rostralis. The arteria cerebri media is the lateral continuation of the ICA via the CAC. This artery courses dorsally through the sulcus rhinalis lateralis, between the lobus piriformis and gyri insulae and sylvii. From the caudolateral surface of the CAC (arteria communicans caudalis), three arteries emerge: the a. cerebri caudalis, the rami choroidei caudalis, and the a. cerebelli rostralis (in order from rostral to caudal). The arteria cerebri caudalis courses deep to the lobus piriformis to supply caudal portions of the cerebrum. The a. cerebri caudalis also courses deep to the lobus piriformis, closer to the cerebellum. Ultimately, this vessel supplies the tectum mesencephali. The arteria cerebelli rostralis branches from the CAC near the pars petrosal of the os temporale, and courses dorsolaterally along the pars rostralis of the cerebellum. The caudal-most branch of the circulus arteriosus cerebri is the arteria basilaris, which forms at the confluence of the arteriae communicans caudalis. The a. basilaris is situated on the midline, along the ventral surface of the metecephalon and myelencephalon, tapering into the a. spinalis ventralis after giving off the a. cerebelli caudalis. The former arteries are the main sources of blood to the cerebellum.

### Hard tissue results: osteological correlates for ruminant cranial arteries

Five hard tissue features were identified that correlate the presence or absence of an a. carotis interna or rete mirabile epidurale. These features correlate directly with the presence of an arterial feature. In most cases, the presence of a correlate for one arterial state is indicative of the absence of that arterial state in the other. This is because artiodactyls effectively replace a patent a. carotis interna with the rete mirabile epidurale. Osteological correlates were sought both internally and externally. The presence and absence of these correlates is summarized in Table 1.

#### Carotid groove, os basisphenoidale

Inside the braincase, the presence of an a. carotis interna leaves a pronounced sulcus caroticus on the internal surface of the os basisphenoidale in *Tragulus*, *Hyemoschus*, *Moschiola*, and *Equus*. This groove deepens throughout development, as the cranial base develops and ossifies in tandem with the development of the cranial arteries (Padget, 1948; O’Brien, 2012). In artiodactyls with a rete mirabile epidurale, such as the domestic goat (*Capra hircus*), this groove is absent.

#### Medial bullar groove

Note: This correlate is only applicable to ruminants. In *Moschiola*, the a. carotis interna leaves a groove on the medial wall of the bulla tympanica as it ascends toward the basicranium. This groove was not identified in the neonate or early juvenile specimens, and can be slight in sub-adults. The groove is most clearly identifiable where it contacts the distal-most extent of the extracranial a. carotis interna. This groove is not identified in *Equus*, as the a. carotis interna and foramen lacerum are positioned well rostral of the bulla.

#### Foramen orbitorotundum

Typically, euungulates that do not possess a rete mirabile epidurale do not join the foramen rotundum with the fissura orbitalis into a “foramen orbitorotundum,” and thus have one more alisphenoid foramen (Sisson & Grossman, 1967). This is exemplified in *Equus*. In ruminants with a rete mirabile epidurale, the rami connecting the a. maxillaris to the rete mirabile epidurale enter the skull through the large, confluent foramen orbitorotundum. Although all ruminants examined possess a foramen orbitorotundum, the morphology of this foramen differed based on presence or absence of an a. carotis interna in tragulids. In *Moschiola*, the foramen orbitorotundum has a pronounced notch on the ventromedial border, due to contact with the a. carotis interna. This notch was found on skulls of all contemporary tragulids. In *Odocoileus* and *Capra*, the foramen orbitorotundum is smooth and accommodates the network of arteries that supply the rete mirabile epidurale.

#### Foramen lacerum

In *Equus* and *Moschiola*, the a. carotis interna enters the braincase through the anterior foramen lacerum. In both cases, the foramen lacerum possess notches that correspond with the a. carotis interna.

#### Foramen ovale

In ruminants with a rete mirabile epidurale, the foramen ovale frequently has a subtle, oblique notch that corresponds to a ramus or rami that supply the rete mirabile epidurale. This notch yields a foramen ovale that is trapezoidal in shape. In the tragulids examined, the foramen ovale lacked such a notch.

## Discussion

Among artiodactyls with described cranial arterial patterns, only the tragulids *T. javanicus* and *T. napu* were previously found to possess a substantial arteria carotis interna. This study documents another example of an artiodactyl without a rete mirabile epidurale, with osteological data suggesting that this condition is common to all tragulids. The use of 3-D anatomical data collection methods, combined with digital dissection, have revealed an extension of previous findings (Fukuta et al., 2007) that may have developmental implications for artiodactyls with and without a rete mirabile epidurale. Developmental studies of prenatal specimens indicate that the a. carotis interna of rete-bearing artiodactyls obliterates during development, such that the brain is not supplied by a major branch of the 3rd aortic arch (Tandler, 1899; Tandler, 1901; Tandler, 1906; Daniel, Dawes & Prichard, 1953; Baldwin, 1964; Gillilan, 1974; Bamel, Dhingra & Sharma, 1975; Wible, 1984). In lieu of a patent a. carotis interna, the brain is largely supplied through the rete mirabile epidurale by the a. maxillaris rostrally (through the foramen orbitorotundum) and the a. pharyngea ascendens caudally (through the foramen ovale, foramen lacerum, or canalis caroticus; see e.g., Daniel, Dawes & Prichard, 1953; Sisson & Grossman, 1967; Smuts & Bezuidenhout, 1987). In several artiodactyls, particularly suids, the a. pharyngea ascendens (or, more appropriately the pharyngocarotid anastomosis *sensu* Wible, 1984) is frequently identified as the a. carotis interna, however embryological studies indicate that the adult vessel is not homologous (Daniel, Dawes & Prichard, 1953; Wible, 1984). In tragulids, the persistent a. carotis interna is likely homologous to that of other vertebrates (Fukuta et al., 2003), and the a. pharyngea ascendens does not hypertrophy.

Overall, tragulids exhibit a cranial arterial pattern that is similar to other mammals, with key exceptions that are relevant to their status as the sister group of ruminants. First, the a. basilaris of non-ruminant mammals is formed at its caudal extent by the merger of contralateral branches of the occipital artery (see e.g.,Hofmann, 1900; Gillilan, 1974; Sisson & Grossman, 1967). This results in a caudal to rostral direction of blood flow through the a. basilaris, such that its parent artery, the a. occipitalis, provides collateral circulation to the CAC (Daniel, Dawes & Prichard, 1953; Gillilan, 1974). In ruminants, now inclusive of tragulids, the a. basilaris does not significantly anastomose with the a. occipitalis, instead diminishing in caliber as it passes caudally, away from the CAC (Daniel, Dawes & Prichard, 1953; Gillilan, 1974; Fukuta et al., 2007; this study). Consequently, blood flows from rostral to caudal through the a. basilaris of ruminants. This morphology has important functional implications in ruminants with a rete mirabile epidurale: the vast majority of blood distributed through the CAC must first pass through the retial heat exchanger, ensuring that nearly all blood bound for the cerebrum and cerebellum is cooled when selective brain cooling is active (Gillilan, 1974).

The second important distinction that can be identified in the arterial pattern of *Moschiola meminna* is the substantial intracranial connection between the a. maxillaris and a. carotis interna. In the typical mammalian condition, the a. maxillaris does not enter the cranial cavity. In *Moschiola*, however, the a. maxillaris gives rise to a ramus that enters the braincase via the foramen orbitorotundum, where it directly anastomoses with the a. carotis interna as it scours the internal surface of the sphenoid (Fig. 4). This anastomosis is typical of non-suiform artiodactyls, which supply the carotid rete through rami from the a. maxillaris (see e.g., Daniel, Dawes & Prichard, 1953; Gillilan, 1974; Sisson & Grossman, 1967). In tragulids, this connection persists throughout the animal’s life; however, in other artiodactyls for which cranial arterial patterns are documented, a substantial a. carotis interna obliterates early in development (Wible, 1984). This intracranial carotico-maxillary anastomosis may be a vital prerequisite for the development and evolution of the rete mirabile epidurale and the efficient selective brain cooling that is facilitated by this vascular feature. Without another substantial influx of oxygenated blood to the brain, the developmental loss of a functional pars extracranialis of the ICA could be deleterious. Thus, it is this aberrant anastomosis that may have enabled the evolution of the artiodactyl rete mirabile epidurale.

In evolutionary terms, the complete, patent a. carotis interna of tragulids has been hypothesized as either a plesiomorphy for ruminants, or an apomorphic feature of tragulids that has arisen due to adaptation (Fukuta et al., 2007). If the tragulid condition is plesiomorphic for ruminants, it suggests that the rete mirabile epidurale may have evolved several times among artiodactyls. If the lack of a rete is an apomorphy for the Tragulidae, then the driving selective or developmental pressures should be investigated further. By surveying the cranial arterial pattern of all extant tragulids, either through soft tissue dissection or identification of hard tissue correlates, the presence of an a. carotis interna can be confirmed throughout the family. Although this finding does not provide direct evidence for either evolutionary hypothesis, it is important to note that extant mouse deer have experienced a diverse suite of selective pressures across their extensive evolutionary history. Not the least of these is habitat, with respect to temperature and hydrological budget. For example, *H. aquaticus*, the water chevrotain, is semi-aquatic and inhabits the dense, equatorial forests of Africa (Dubost, 1978). The Asian taxa, *Tragulus* and *Moschiola*, are found in more hydrologically variable environments, with some species preferring more open, drier habitats and others preferring dense vegetation and more moist habitats (Pickford, Senut & Mourer-Chauviré, 2004; Groves & Meijaard, 2005). From a temporal perspective, divergence between the genera *Tragulus* and *Hyemoschus* likely occurred in the early Miocene (Hassanin et al., 2013), as the ancestor of *Hyemoschus* entered the African landmass pursuant to the formation of connections between Europe and Africa (Pickford, 2001; Sanchez et al., 2010). The geographic, ecological, and temporal segregation between living tragulids should result in different suites of selective pressures. If the lack of a rete mirabile epidurale is an apomorphy among tragulids, this condition was almost certainly established prior to the separation of modern groups. Overall, both this study and that of Fukuta and colleagues (2007) emphasize the need for additional developmental and evolutionary studies into the origins of ruminant cranial vascular patterns.

## Additional Information and Declarations

### Data Availability

The following information was supplied regarding data availability: FigShare:
http://figshare.com/s/e22fde06688711e5babe06ec4bbcf141
http://figshare.com/s/f1f3cb9a688711e5ab6506ec4bbcf141.
